# Evaluating Performance Among National Basketball Association Players After Jones Fractures: A Retrospective Cohort Study

**DOI:** 10.1177/23259671241300330

**Published:** 2024-12-11

**Authors:** Andrew H. Kim, Myung-Jin Cha, Alvina Pan, Gianna Dafflisio, Miguel A. Cartagena-Reyes, Frederick Mun, Jonathan R. Kaplan, Amiethab A. Aiyer

**Affiliations:** † Department of Orthopaedic Surgery, Johns Hopkins University, Baltimore, Maryland, USA; ‡ Department of Orthopaedics and Rehabilitation, Penn State Milton S. Hershey Medical Center, Hershey, Pennsylvania, USA; § Department of Orthopaedic Surgery, Duke University Medical Center, Durham, North Carolina, USA; Investigation performed at Johns Hopkins University, Baltimore, Maryland, USA

**Keywords:** Jones fracture, National Basketball Association, stress fracture, basketball, metatarsal, foot

## Abstract

**Background:**

In professional basketball, Jones fractures are among the most common cause of lower extremity stress injury. Despite its prevalence, there is a paucity of research on the impact of Jones fractures on athletic performance in the National Basketball Association (NBA).

**Purpose:**

To determine the impact of Jones fractures on return to play and performance among NBA players when compared with preinjury values and healthy matched controls.

**Study Design:**

Cohort study; Level of evidence, 3.

**Methods:**

NBA injury reports were analyzed to identify 18 players who sustained a Jones fracture between 2011 and 2022. Reports were verified through public press releases, social media accounts, and player profiles. A publicly available database was used to collect player data including position, age, and years of NBA experience. Performance and advanced statistics were collected for seasons pre- and postinjury. Players were 1:1 matched with healthy controls based on age, player position, and career performance. Univariate and multivariable regression analyses were performed to compare advanced and per-game performance statistics between injured and healthy control players.

**Results:**

NBA players who sustained a Jones fracture had a mean age of 23.9 ± 2.31 years at the time of injury. The mean NBA experience was 3.00 ± 2.35 years and mean preinjury minutes played per game was 19.64 ± 11.24. All players (18/18) were able to return to NBA-level competition the year following the injury. There was no change in player efficiency rating (PER), value added, and estimated wins added metrics when comparing pre- and postinjury performances among injured players. Injured players missed more games (*P* < .001) postinjury compared with the matched controls. Multivariate analysis revealed that for players with a height of <2 m, every additional centimeter of height significantly decreased postinjury PER by 1.08 (95% CI, 0.35-1.80; *P* < .01).

**Conclusion:**

Despite its severity, most NBA players who sustain Jones fractures can return to preinjury performance and level of competition. There was no statistically significant decline in advanced statistics in the following season after injury, with a significant decrease in games played when comparing injured players with their healthy controls.

Basketball is a physically demanding sport, posing significant risk of lower extremity injury to athletes through high-impact repetitive movements.^3,24,31^ This is especially true of National Basketball Association (NBA) players, with lower extremity injuries accounting for nearly 62.4% of all injuries.^9^ Fifth-metatarsal base fractures are difficult to manage, especially when they involve zone 2 or zone 3, which represent vascular watershed areas,^25^ resulting in higher rates of refracture or nonunion among elite athletes.^14,19^ Zone 2 or Jones fractures have the highest frequency of operative intervention among lower extremity injuries in athletes.^18^ Thus, there is significant concern on how management of Jones fractures may affect return to play and long-term performance among NBA players.

While many studies have examined the impact of Jones fracture on return to play among NBA players,^4,18,27,29^ minimal research has evaluated the impact of Jones fracture on in-game performance statistics.^4,29^ The purpose of this study was to provide an updated overview (1) examining preinjury and postinjury performances and advanced statistics among NBA players who sustained a Jones fracture and (2) comparing their preinjury and postinjury statistics with healthy 1:1 matched controls through the 2011-2012 and 2021-2022 seasons.

## Methods

### Data Source

Using previously verified methods,^1,2,5,13,16,22,23^ NBA players who sustained a Jones fracture over a 10-year period through the 2011-2012 and 2021-2022 seasons were identified using a publicly available, comprehensive, online injury database (http://www.prosportstransactions.com).

### Inclusion and Exclusion Criteria

Inclusion criteria were players undergoing surgical or conservative treatment for primary diagnoses reported as “metatarsal fracture,”“foot fracture,”“Jones fracture,” and “toe fracture.” Injury reports were verified through public press releases, transaction reports, social media accounts, and player profiles to confirm the diagnosis of a Jones fracture. Exclusion criteria were players who had a primary diagnosis of “unspecified stress fracture of foot” or “unspecified stress reaction of foot” that could not be verified as a Jones fracture, players who did not sustain a Jones fracture, players who did not play an NBA game pre- or postinjury, and players who never played in the NBA.

### 1:1 Matching

Eighteen players included for analysis were then matched to healthy controls based on similarity scores provided by a comprehensive online database (http://www.basketballreference.com). Similarity scores are based on a player’s career length, career statistical performance, and position played. Among healthy control players, the index year was matched to the year of injury of their matched participant.

### Player and Performance Variables

Player profiles were utilized to record demographic and player information, including height, weight, body mass index (BMI), age, position, and years of NBA experience prior to injury. NBA players were then assigned to backcourt or frontcourt position designations if they played point guard and shooting guard or small forward, power forward, and center, respectively. Player performance variables and advanced statistics were collected for each player 1 season preinjury and 1 season postinjury using the official website of the NBA (http://www.nba.com) and ESPN (Entertainment and Sports Programming Network) (http://espn.go.com/nba/). Performance variables collected included games missed, minutes played per game, and points per game. Additional variables were collected per 36 minutes played to account for variations in playing time and included assists, rebounds, steals, and blocks. Advanced statistics included player efficiency rating (PER), value added (VA), and estimated wins added (EWA). PER is an objective, standardized metric of a player’s per-minute productivity that is pace-adjusted by team.^12^ The PER is normalized to a league mean of 15.00 every season and has been utilized by numerous studies to analyze postinjury outcomes within the NBA.^1,4,5,22,34^ VA is the estimated number of points a player adds to his team’s season total, and EWA is the estimated number of wins a player adds to his team’s season total when compared with a “replacement-level” player.^12^ Return-to-play rates were also recorded and were defined as playing ≥1 game at the NBA level in the following season after injury. This definition has been previously utilized in orthopaedic studies examining the return-to-play rate among NBA players.^8,15,20^

The data analyzed in this study were derived from publicly accessible databases and did not require institutional review board approval.

### Statistical Analysis

Demographic variables and pre- and postinjury performance metrics were summarized using mean and standard deviation or median and interquartile range (25th-75th percentiles) and compared between injured and healthy control players using Wilcoxon rank-sum tests.^33^ Pre- and postinjury performance was compared among injured players using Wilcoxon matched-pairs signed-rank test. A series of multivariable linear regression models were fit to the data to assess predictors of postinjury PER measure among injured players. The models included cluster-robust variance estimates for each individual player to account for repeated observations for some players.^28^ The shape of the relationship between continuous predictors (such as age, height, BMI, duration of NBA experience preinjury, minutes played preinjury, and preinjury PER) and postinjury PER were explored using visual tools and appeared linear. Linear splines were added to represent a change in linear slope at a certain value of a continuous predictor (ie, knot).^10^ In model 1, all numeric variables were linear for ease of interpretation. To explore additional nonlinearities for minutes played per game preinjury and player height, models 2 and 3 were created respectively using linear splines. The statistical analyses were performed using STATA statistical software program (Release 18; StataCorp LLC), and significance level was set at *P* < 0.05.

## Results

Twenty-nine players met inclusion criteria for this study. After application of exclusion criteria, 11 players were excluded, with 8 players not having played a single NBA game preinjury, 2 players having not played a single NBA game postinjury, and 1 player having revision screw replacement for a Jones fracture sustained while playing in college. A total of 18 players remained for analysis. Each player had NBA experience prior to sustaining a Jones fracture, with 100% (18/18) able to return to play the following season. Among the 18 players included for analysis, 3 sustained a subsequent Jones fracture after the initial injury. Of total players, 94% (17/18) underwent surgical fixation, with 16.7% (3/18) requiring reoperation. Mean age at the time of injury was 23.94 ± 2.31 years, with a mean NBA experience time of 3.00 ± 2.35 years prior to injury (Table 1).

**Table 1:** Demographic Data for NBA Players With Jones Fractures and Healthy Control Players^*a*^

Variable	Control	Injured	*P*
Age, y	23.94 (2.31)	23.72 (2.05)	.76
Height, cm	201.83 (8.90)	201.56 (8.15)	.92
NBA experience, y	3.00 (2.35)	2.28 (2.05)	.33
Minutes per game	19.64 (11.24)	18.12 (9.18)	.66
Position played, n			>.99
Frontcourt^*b*^	12	12	
Backcourt^*c*^	6	6	

a Data presented during preinjury and preindex seasons as mean (SD) unless otherwise indicated. NBA, National Basketball Association.

b Frontcourt, small forward, power forward, or center.

c Backcourt, point guard or shooting guard.

Players were matched using similarity scores based on performance and position played. Injured and healthy control players did not differ with respect to positions played, with 12 frontcourt and 6 backcourt players. There were no significant differences in age (23.94 ± 2.31 vs 23.72 ± 2.05 years; *P* = .76), height (201.83 ± 8.90 vs 201.56 ± 8.15 cm; *P* = .92), years of NBA experience (3.00 ± 2.35 vs 2.28 ± 2.05 years; *P* = .33), or preinjury minutes played per game (19.64 ± 11.24 vs 18.12 ± 9.18 minutes; *P* = .66) between healthy control and injured players (Table 1).

When comparing preinjury performance between healthy control and injured NBA players, injured players missed significantly more games at baseline compared with healthy players (21.5 vs 0.0; *P* < .001) (Table 2).

**Table 2:** Comparison of Preinjury Performance Between Healthy Controls and Injured NBA Players^*a*^

Variable	Control (n = 18)	Injured (n = 18)	Total (N = 36)	*P*
Games missed	0.0 (0.0-1.0)	21.5 (8.0-40.0)	3.5 (0.0-21.5)	<.001
Minutes per game	17.5 (11.3-20.9)	21.2 (12.2-27.2)	18.3 (11.8-25.3)	.55
Assists^*b*^	2.13 (1.79-4.38)	1.65 (1.44-2.54)	1.97 (1.60-3.11)	.11
Rebounds^*b*^	6.94 (4.67-10.1)	6.16 (3.92-7.81)	6.75 (4.08-8.57)	.61
Steals^*b*^	1.36 (1.07-1.92)	1.16 (0.81-1.48)	1.25 (0.87-1.68)	.30
Blocks^*b*^	0.57 (0.27-1.47)	0.65 (0.25-0.94)	0.62 (0.26-1.00)	.73
Points per game	5.60 (4.00-8.60)	7.60 (2.90-11.30)	6.35 (3.90-10.8)	.53

a Values expressed as median (interquartile range). NBA, National Basketball Association.

b Performance variables per 36 minutes played.

When comparing postinjury performance between healthy control and injured players, injured players had a significantly larger number of games missed (50.0 vs 0.0; *P* < .001), with no significant difference in minutes per game, points per game, or in-game statistics (Table 3).

**Table 3:** Comparison of Postinjury Performance Between Healthy Controls and Injured NBA Players^*a*^

Variable	Control (n = 18)	Injured (n = 18)	Total (N = 36)	*P*
Games missed	0.0 (0.0-3.0)	50.0 (19.0-61.0)	12.0 (0.0-50.0)	<.001
Minutes per game	20.0 (10.4-24.4)	15.7 (10.0-30.2)	16.6 (10.2-28.0)	.76
Assists^*b*^	2.57 (1.73-4.24)	2.05 (1.01-3.86)	2.17 (1.42-4.15)	.25
Rebounds^*b*^	6.73 (4.41-9.30)	7.33 (4.24-8.41)	6.96 (4.32-8.90)	.92
Steals^*b*^	1.36 (1.05-1.59)	1.21 (0.53-1.35)	1.30 (0.86-1.50)	.09
Blocks^*b*^	0.61 (0.29-1.60)	0.51 (0.24-1.41)	0.56 (0.25-1.50)	.59
Points per game	6.95 (3.4-8.7)	5.90 (3.0-14.4)	6.65 (3.2-11.5)	.96

a Values expressed as median (interquartile range). NBA, National Basketball Association.

b Performance variables based on per 36 minutes played.

Performance variables and advanced statistics were analyzed among NBA players who sustained a Jones fracture. Injured players missed a significantly greater number of games postinjury when compared with preinjury (50.0 vs 21.5; *P* = .02). In the following season after injury, players played a mean 39.2 ± 29.0 games. There were no significant differences in minutes per game, points per game, in-game statistics, or advanced statistics between pre- and postinjury states (Table 4).

**Table 4:** Comparison of Pre- and Postinjury Performance and Advanced Statistics Among Injured NBA Players^*a*^

Variable	Preinjury (n = 18)	Postinjury (n = 18)	*P*
Games missed	21.5 (8.0 to 40.0)	50.0 (19.0 to 61.0)	.02
Minutes per game	21.15 (12.2 to 27.2)	15.65 (10.0 to 30.2)	.30
Assists^*b*^	1.65 (1.44 to 2.54)	2.05 (1.01 to 3.86)	.87
Rebounds^*b*^	6.16 (3.92 to 7.81)	7.34 (4.24 to 8.41)	.77
Steals^*b*^	1.16 (0.81 to 1.48)	1.21 (0.53 to 1.35)	.44
Blocks^*b*^	0.65 (0.25 to 0.94)	0.51 (0.24 to 1.41)	.43
Points per game	7.6 (2.9 to 11.3)	5.9 (3.0 to 14.4)	.13
PER	13.43 (9.87 to 19.33)	12.77 (8.82 to 14.04)	.15
VA	0.75 (–2.3 to 38.4)	11.15 (0.0 to 32.4)	.14
EWA	0.00 (–0.1 to 1.3)	0.35 (0.0 to 1.1)	.14

a Values expressed as median (interquartile range). EWA, estimated wins added; NBA, National Basketball Association; PER, player efficiency rating; VA, value added.

b Performance variables per 36 minutes played.

Multivariate regression analysis was performed for postinjury performance using PER among injured NBA players (Table 5). In model 1, all numeric variables were assumed to have a linear relationship with postinjury PER. This model demonstrated that no variables were associated with postinjury PER after adjusting for age, height, BMI, minutes per game, and years of NBA experience. In model 2, a nonlinear relationship between minutes per game and postinjury PER was assumed. This model demonstrated no association between minutes per game and PER up to 25 minutes played. After 25 minutes played, however, for every additional minute played, there was a significant increase of 1.53 in postinjury PER (95% CI, 0.35-2.71; *P* < .05) after adjusting for age, height, BMI, preinjury PER, and years of NBA experience. In model 3, height and minutes per game were assumed to have a nonlinear relationship with postinjury PER. For every additional minute played after 25 minutes, postinjury PER significantly increased by 1.54 (95% CI, 0.63-2.45; *P* < .01) after adjusting for age, height, BMI, preinjury PER, and years of NBA experience. With regard to height <2 m, every additional centimeter of height significantly decreased postinjury PER by 1.08 (95% CI, 0.35-1.80; *P* < .01) after adjusting for age, minutes per game, BMI, preinjury PER, and years of NBA experience.

**Table 5:** Multivariable Linear Regression Models for Postinjury Performance Using PER Measure Among Injured NBA Players^*a*^

	Model 1	Model 2	Model 3
PER prior to injury	0.294 [–0.182, 0.770]	0.0295 [–0.401, 0.461]	0.0589 [–0.272, 0.390]
Age at time of injury	–2.226 [–5.850, 1.397]	–0.536 [–3.702, 2.631]	0.591 [–1.986, 3.168]
Height	–0.108 [–0.577, 0.362]	–0.217 [–0.599, 0.165]	
Height, <200			–1.075^*c*^ [–1.800, –0.350]
Height, ≥200			0.462 [–0.139, 1.063]
BMI	0.950 [–0.830, 2.730]	1.175 [–0.247, 2.597]	1.514^*b*^ [0.394, 2.635]
Minutes played per game preinjury	0.114 [–0.373, 0.601]		
≤25 minutes played per game preinjury		–0.419 [–0.990, 0.152]	–0.318 [–0.762, 0.127]
>25 minutes played per game preinjury		1.529^*b*^ [0.346, 2.712]	1.539^*c*^ [0.633, 2.445]
Years of NBA experience preinjury	2.326 [–1.546, 6.199]	1.219 [–1.973, 4.410]	0.168 [–2.408, 2.743]
Intercept	49.32 [–78.76, 177.4]	38.86 [–62.97, 140.7]	169.9^*b*^ [42.08, 297.7]
N	18	18	18
AIC	127.8	119.2	109.2
*R* ^2^, %	52	73	86

a Values expressed as estimated slope [95% CI] unless otherwise indicated. AIC, Akaike information criterion; BMI, body mass index; NBA, National Basketball Association; PER, player efficiency rating.

b *P* < .05.

c *P* < .01.

## Discussion

Although Jones fractures significantly affected the number of games missed when compared with healthy controls, our study found that NBA players experienced no significant decline in advanced statistical metrics, minutes, steals, assists, rebounds, blocks, and points per game when comparing preinjury with postinjury performance. Additionally, despite a high reoperation rate (16.7%), 100.0% (18/18) of players were able to return to NBA-level of play the following season. Our study also found significant relationships between player height and postinjury PER.

When compared with healthy control players, NBA players who sustained a Jones fracture experienced a significant increase in games missed preinjury (0.0 vs 21.5; *P* < .001) and postinjury (0.0 vs 50.0; *P* < .001). The greater number of games missed in the following season among injured players may be attributed to scheduled days of rest to improve game performance^30^ and minimize the risk of reinjury.^32^ Khan et al^18^ also found that among NBA players who experienced a lower extremity stress injury or fracture, the number of games played (72.0 vs 61.3) and steals per game (0.9 vs 0.8) significantly decreased upon return to play. While our study found a significant difference in the number of games missed preinjury compared with postinjury (21.5 vs 50.0; *P* = .02) among players who sustained a Jones fracture, there was no significant difference in steals per game (1.16 vs 1.21; *P* = .44). Although a similar study examining the impact of Jones fractures on NBA performance between the 1994-1995 and 2012-2013 seasons found a significant decline in points and assists per game among injured players, this may be the result of their cohort’s having a greater percentage of backcourt players.^4^

Although the literature has demonstrated the detrimental impact of Achilles tendon^1,6,17^ and anterior cruciate ligament^4,11,26^ injuries on career length and performance in the NBA, our study found no significant decline in postinjury advanced statistics or in minutes, steals, assists, rebounds, blocks, and points per game in NBA players who sustained a Jones fracture. These findings align with Begly et al^4^ and Singh et al,^29^ who similarly found no significant difference in change in 1-year PER pre- and postinjury.^4,28^ Additionally, Choudhry et al. found no significant difference in minutes (23.3 vs 21.6 minutes), assists (1.8 vs 1.7), rebounds (0.4 vs 3.8), blocks (0.6 vs 0.5), and points per game (9.6 vs 8.8) pre- and postinjury among NBA players sustaining foot fracture injuries.^7^ Despite concern on how Jones fractures may affect return to play and long-term performance, our results suggest that fractures to the fifth metatarsal do not significantly impair return-to-play rates and have little impact on performance the following season after injury.

While previous studies have reported that NBA players with higher preinjury PER had significantly higher postinjury PER,^4,18^ our study also utilized multivariable nonlinear regression analysis, which found that each additional minute played after 25 minutes resulted in a postinjury PER increase of 1.53. These findings are intuitive given that NBA players who performed at greater levels preinjury are more likely to perform at greater levels and play >25 minutes postinjury. Our study also found that with regard to player height <2 m, every additional centimeter resulted in a 1.08 decrease in postinjury PER. This may suggest a negative trade-off between height and postinjury PER among NBA point guards and shooting guards who have sustained a Jones fracture.

### Limitations

Our study is not without limitations. First, although the methods performed in this study are consistent with prior studies, inaccessibility to official NBA medical records prevents consideration of concomitant injuries and fracture severity preinjury, confounding factors that could affect postinjury performance metrics. Second, given the retrospective nature of our study and limited sample size, our cohort is subject to selection and observer bias. Additionally, the size of our sample prevented position-specific analysis. The lack of power also makes our study susceptible to type 2 error. Given the way data acquisition was performed, information regarding surgical invasiveness, techniques, rehabilitation protocols, union rates, and surgical complications were unavailable. These confounding variables may have significantly affected prognosis, performance, and return to play. This is further compounded by the discordance in management strategy for Jones fractures among foot and ankle surgeons.^21^ Although variations in treatment regimens and protocols were likely observed over the collection period, this time frame was selected to analyze novel data while maximizing our sample size. Finally, reasons for failure to return to play or declines in performance and advanced statistics are multifactorial. They could be the result of financial or personal issues or changes in team utilization, roster compositions, or coaching changes.

## Conclusion

Despite the high rates of nonunion, refracture, and reoperation among elite athletes, NBA players who undergo operative intervention for Jones fractures can return to play without a statistically significant impact on overall performance. Our study demonstrates that despite a high reoperation rate, NBA players analyzed between the 2011-2012 and the 2021-2022 seasons exhibited no statistically significant decline in advanced statistics or performance metrics in the following season after sustaining a Jones fracture.
